# Editorial: Advances in metabolism and chemodiversity - focus - plant enzymes

**DOI:** 10.3389/fpls.2023.1227424

**Published:** 2023-06-16

**Authors:** Zhi-Yan Du, Yang Qu, Zhenhua Liu, Mariam Gaid

**Affiliations:** ^1^ Department of Molecular Biosciences and Bioengineering, University of Hawaii at Manoa, Honolulu, HI, United States; ^2^ Department of Chemistry, University of New Brunswick, Fredericton, Canada; ^3^ Shanghai Collaborative Innovation Center of Agri-Seeds, School of Agriculture and Biology, Shanghai Jiao Tong University, Shanghai, China; ^4^ Institute of Pharmaceutical Biology, Technische Universität Braunschweig, Braunschweig, Germany

**Keywords:** photosynthetic organisms, enzyme activity, stress response, metabolic engineering, metabolism

Plant enzymes are indispensable for plant metabolism and are critical determinants of the extensive chemodiversity observed in plants. These enzymes serve as primary catalysts in biosynthetic pathways, enabling the biosynthesis of a diverse range of secondary metabolites, such as alkaloids, terpenes, and phenolics. These metabolites are essential for the distinctive sensory qualities of plants, e.g., flavors, scents, and colors, and they also possess significant biological activities with promising applications in agriculture, medicine, and industry. Recent scientific investigations have been devoted to unraveling the intricate biochemistry of enzymes in photosynthetic organisms, elucidating their catalytic mechanisms, regulatory processes, and evolutionary trajectories. For instance, recent researches have elucidated dynamic diversifying mechanisms underlying the synthesis of phenolic acid in *Salvia miltiorrhiza* (Zhou et al., 2021), alkaloids in *Catharanthus roseus* (Eng et al., 2022), triterpenes in *Eriobotrya japonica* (Su et al., 2021), and xanthones in *Hypericum* species (Singh et al., 2020; Singh et al., 2021). These studies have yielded valuable insights into the biosynthetic pathways of pivotal compounds in non-model plants. By augmenting the understanding of plant enzyme biochemistry, scientists can harness their immense potential for advancing sustainable agriculture, facilitating drug discovery, and fostering the development of plant-based products, thereby endrosing positive impacts on human health and the environment.

This Research Topic includes six original research articles, with a special focus on the function of plant enzymes. Zhang et al. reported that *Prunus mume* cinnamyl alcohol dehydrogenase 1 and 2 (*Pm*CAD1 and 2) are the major contributors to the cinnamyl alcohol biosynthesis and emission, which was identified after analysing the endogenous volatile compounds and the transcriptomes gleaned from six *P. mume* cultivars (**Figure 1A**). Another research on floral fragrant components in *Rosa rugosa* revealed 156 differential volatile organic compounds, from two metabolic pathways: the monoterpenoid biosynthetic pathway and the amino acid (phenylalanine, tyrosine, and tryptophan) biosynthesis pathway, which are important for further genetic engineering of floral metabolites and the breeding of new rose cultivars (**Figure 1B**) (Cheng et al.). In the herbaceous plant *Salvia miltiorrhiza*, *SmDXS5* encoding 1-deoxyxylulose 5-phosphate synthase 5 was found as a ‘ molecular valve’ that is important for the regulation of primary and secondary metabolic flow of tanshinones in *S. miltiorrhiza* (**Figure 1C**) (Zhang et al.). Interestingly, the increased terpenoid levels by *SmDXS5* overexpression is accompanied by a reduction of the phenylalanine ammonia lyase and contents of phenolics. In the boraginaceous plant *Arnebia euchroma*, *AeHGO*, a gene belonging to the cinnamyl alcohol dehydrogenase family catalyzes a reversible alchohol oxidation reaction and divert the shikonin biosynthesis toward the formations of shikonofurans. The gene was identified from coexpression analyses of transcriptome data sets of shikonin-proficient and shikonin-deficient cell lines of *A. euchroma* and it can be used for metabolic engineering of shikonin derivatives (**Figure 1D**) (Wang et al.). Wangpaiboon et al. demonstrated *Manihot esculenta* pullulanase (*Me*PUL) and cassava isoamylase 3 (*Me*ISA3) synergistically debranched *β*-limit dextrin, a major starch catabolising process in dicots. The finding suggests an important role of these two enzymes in cassava starch catabolism (**Figure 1E**). *Scirpus planiculmis* is known as a common weed found in the cotton field, which can cause stress and yield loss to the cotton plants. The research by Zhang et al. revealed the mechanism of physiological response in cotton plants impacted by *S. planiculmis* by field competition herbicide mediation experiments (**Figure 1F**).

**Figure 1:**
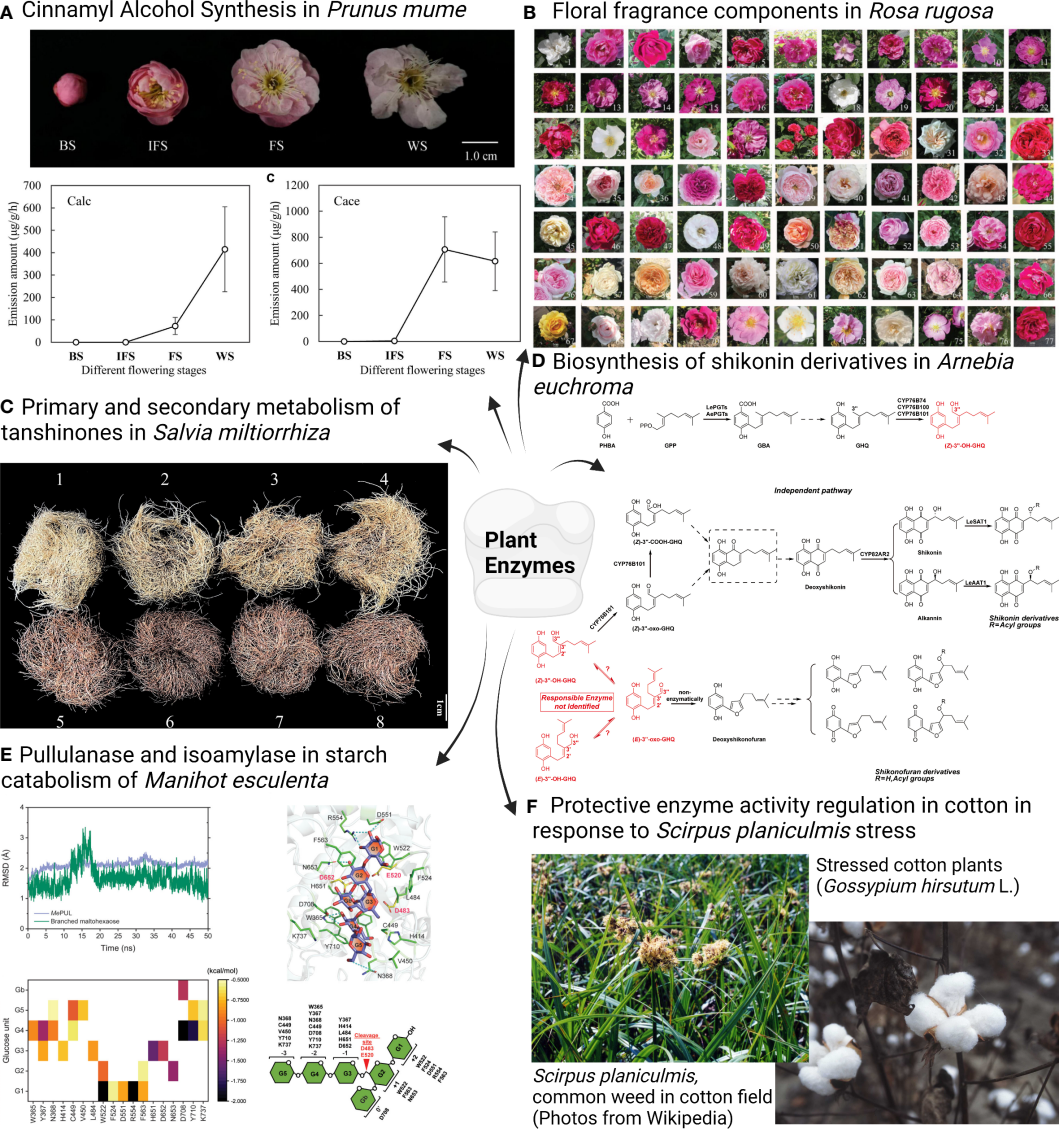
Overview of the original research articles in this Plant Enzyme Research Topic. **(A)** Cinnamyl Alcohol Synthesis in *Prunus mume* (Siebold) Siebold & Zucc. **(B)** Floral fragrance components in *Rosa rugosa* Thunb. **(C)** Primary and secondary metabolism of tanshinones in *Salvia miltiorrhiza* Bunge. **(D)** Biosynthesis of shikonin derivatives in *Arnebia euchroma* (Royle) I.M. Johnst. **(E)** Pullulanase and isoamylase in starch catabolism of *Manihot esculenta* Crantz. **(F)** Protective enzyme activity regulation in cotton in response to stress of *Scirpus planiculmis* F. Schmidt.

The collection of articles in this Research Topic demonstrates the significance of plant enzymes in various biological processes and applications, and the findings can contribute to the construction of genetically-engineered plants as the future sources of diverse bioproducts with better agricultural traits.

## Author contributions

All authors listed have made a substantial, direct, and intellectual contribution to the work and approved it for publication.
